# Neurotensin Receptor 1 Is Expressed in Gastrointestinal Stromal Tumors but Not in Interstitial Cells of Cajal

**DOI:** 10.1371/journal.pone.0014710

**Published:** 2011-02-18

**Authors:** Petra Gromova, Brian P. Rubin, An Thys, Christophe Erneux, Jean-Marie Vanderwinden

**Affiliations:** 1 Laboratory of Neurophysiology, Faculty of Medicine, Université Libre de Bruxelles (ULB), Brussels, Belgium; 2 Anatomic Pathology and Molecular Genetics, Cleveland Clinic, Lerner Research Institute and Taussig Cancer Center, Cleveland, Ohio, United States of America; 3 Institut de Recherche Interdisciplinaire en Biologie Humaine et Moléculaire (IRIBHM), Faculty of Medicine, Université Libre de Bruxelles (ULB), Brussels, Belgium; Katholieke Universiteit Leuven, Belgium

## Abstract

Gastrointestinal stromal tumors (GIST) are thought to derive from the interstitial cells of Cajal (ICC) or an ICC precursor. Oncogenic mutations of the KIT or PDGFRA receptor tyrosine kinases are present in the majority of GIST, leading to ligand-independent activation of the intracellular signal transduction pathways. We previously investigated the gene expression profile in the murine *Kit* ^K641E^ GIST model and identified *Ntsr1* mRNA, encoding the Neurotensin receptor 1, amongst the upregulated genes. Here we characterized *Ntsr1* mRNA and protein expression in the murine *Kit* ^K641E^ GIST model and in tissue microarrays of human GIST. *Ntsr1* mRNA upregulation in *Kit* ^K641E^ animals was confirmed by quantitative PCR. Ntsr1 immunoreactivity was not detected in the Kit positive ICC of WT mice, but was present in the Kit positive hyperplasia of *Kit* ^K641E^ mice. In the normal human gut, NTSR1 immunoreactivity was detected in myenteric neurons but not in KIT positive ICC. Two independent tissue microarrays, including a total of 97 GIST, revealed NTSR1 immunoreactivity in all specimens, including the KIT negative GIST with PDGFRA mutation. NTSR1 immunoreactivity exhibited nuclear, cytoplasmic or mixed patterns, which might relate to variable levels of NTSR1 activation. As studies using radio-labeled NTSR1 ligand analogues for whole body tumor imaging and for targeted therapeutic interventions have already been reported, this study opens new perspectives for similar approaches in GIST.

## Introduction

Gastrointestinal stromal tumors (GIST) are the most common sarcoma of the gastrointestinal tract. They are thought to derive from the interstitial cells of Cajal (ICC) or an ICC precursor. Approximately 85% of GIST harbor oncogenic *KIT* mutations and 7% contain oncogenic platelet-derived growth factor receptor alpha (*PDGFRA*) mutations. KIT and PDGFRA are members of the type III receptor tyrosine kinase family. Oncogenic mutation of KIT or PDGFRA lead to constitutive autophosphorylation, ligand-independent activation of the downstream signal transduction pathways and subsequent deregulation of cell proliferation, survival and migration [1], [2].

Since the release of imatinib mesylate (a.k.a. STI571, Gleevec™), an inhibitor of KIT and PDGFRA activation, the immunohistochemical demonstration of KIT immunoreactivity (-ir) has been essential for the diagnosis of GIST [3]. However, up to 15% of GIST show weak or negative staining for KIT [4]. Many of these “KIT-negative” GIST harbor *PDGFRA* mutations and may respond to imatinib treatment [5], [6], hence need to be classified correctly. Recently, Discovered on GIST-1 (DOG1, a.k.a *anoctamin*, ANO1), Protein kinase C theta (PKC theta) and Carbonic anhydrase II (CAII) have been suggested as additional diagnostic markers and/or putative therapeutic targets [7]–[10]. Noteworthy, these genes are also expressed in normal KIT-ir ICC [7], [10], [11] and belong to the gene expression profile of KIT-ir ICC in the mouse small intestine [12]. Their presence in GIST thus likely reflects the fact that these tumors arise from the lineage of KIT-ir ICC.

We have recently identified genes expressed in GIST, but not in normal KIT-ir ICC, by comparing gene expression profiles of the antrum of transgenic mice harboring the oncogenic *Kit* K641E mutation [13] and WT littermates, using cDNA microarray. The G-protein-coupled receptor (GPCR), *Neurotensin receptor 1 (Ntsr1)* appeared highly upregulated in *Kit* ^K641E^ antrum [14], while it did not belong to the gene expression profile of normal Kit-ir ICC in the mouse small intestine [12]. As studies using radio-labeled NTSR1 ligand analogues have already been reported for whole body tumor imaging and for therapeutic interventions [15], NTSR1 appeared as an interesting prospect. Hence we have characterized Ntsr1 expression in the murine *Kit* ^K641E^ GIST model, in the normal human gut wall and in human GIST. NTSR1-ir was not detected in normal KIT-ir ICC, but was present in all GIST investigated, including KIT negative GIST harboring PDGFRA mutations.

## Materials and Methods

### The Kit^ K641E^ murine model of GIST

A knock-in *Kit* ^K641E^ murine model of GIST used in this study has been previously described [13]. Two weeks old (P14) *Kit* ^K641E/K641E^, *Kit* ^WT/K641E^ and *Kit* ^WT/WT^ littermates, as well as adult (3–6 month old) *Kit* ^WT/K641E^ and *Kit* ^WT/WT^ animals, were used. Mice were bred and experiments performed in accordance with the ethics committee for animal well-being of the Faculty of Medicine, Université Libre de Bruxelles, Brussels, Belgium.

#### RNA Extraction and PCR

Total RNA was extracted from mouse antrum using RNeasy Mini Kit (Qiagen, Valencia, CA) according to the manufacturer's instructions as described [14]. Genomic DNA was removed with the DNA-free™ kit (Ambion, Inc., Austin, Texas). RNA (1 µg) was reverse transcribed with 200 units of M-MLV Reverse Transcriptase (Invitrogen, Eugene, Oregon) in a reaction containing random primers (Amersham Bioscience, Piscataway, NJ). For reverse transcription PCR (RT-PCR) the cDNA reverse transcription product was amplified with specific primers (Table 1) using 1U Taq DNA polymerase (Qiagen, Valencia, CA) according to the manufacturer's instructions, during 40 cycles of 94°C for 30 sec, 57°C for 45 sec and 72°C for 45 sec. PCR products were separated on 2% agarose gel, stained with ethidium bromide and photographed under UV illumination. Real time quantitative PCR (qPCR) was performed as described [14], using SYBR Green chemistry on a 7500 Real time PCR system (Applied Biosystems, Foster City, CA). Identical conditions, namely 95°C for 10 min; then 40 cycles of 95°C for 15 sec and 60°C for 1 min were used for all primer sets (Table 1). Transcriptional quantification relative to *Gapdh* and *β-actin* housekeeping genes was performed using Qbase™ software [16]. Data were reported as the mean of three independent experiments and presented as normalized relative quantities (NRQ). Statistical analysis was performed in Microsoft Office Excel™ 2003 using unpaired Student's t-test. Differences were regarded as statistically significant when p≤0.05.

**Table 1:** List of primers.

**Primers for RT-PCR (human sequence)**		
NM_006183.3	NT/N F	5′-AGGGCTTTTCAACACTGGGAG- 3′
	NT/N R	5′-TCATACACGTGCCGTTTCAGA- 3′
NM_002531.2	NTSR1 R	5′-GTTGATGGTGGAGCTGACGTAG- 3′
	NTSR1 F	5′-GGATGAGCAGTGGACTCCGT- 3′
NM_001101.3	β-ACTIN F	5′-CATCCACGAAACTACCTTCAACTCC- 3′
	β-ACTIN R	5′-GAGCCGCCGATCCACAC- 3′
**Primers for q-PCR (murine sequence)**		
NM_018766.2	Ntsr1 F	5′-CAAGGTCGTCATCCAGGTTAACAC- 3′
	Ntsr1 R	5′-CCATGACGGTCAGTTTGTTGG- 3′
NM_008747.2	Ntrs2 F	5′-CTCAGAGCCATCGTGGCTGT- 3′
	Ntsr2 R	5′-GAAGACACGGCGTTGTAGAGGA- 3′
NM_019972.2	Ntsr3 F	5′-GAGACAAATGCCAAGGTGGG- 3′
	Ntsr3 R	5′-ACTTGGAATTCTGCTTTGTGGG- 3′
NM_008084.2	Gapdh F	5′-TGTGTCCGTCGTGGATCTGA- 3′
	Gapdh R	5′-CCTGCTTCACCACCTTCTTGA- 3′
NM_007393.3	β-actin F	5′-AACCGTGAAAAGATGACCCAGAT- 3′
	β-actin R	5′-GCCTGGATGGCTACGTACATG- 3′

#### Immunofluorescence staining (IF)

Specimens were processed as described [14]. Briefly, slides were brought to RT, rinsed in 10mM Tris (Merck-Belgolabo, Overijse, Belgium) and 0.15M sodium chloride, pH 7.4 TBS, containing 0.1% (v/v) Triton-X 100 (TBS-TX), and incubated for 1 hour in 10% normal horse serum (NHS) (Hormonologie Laboratoire, Marloie, Belgium) and TBS-TX to reduce background staining. The slides were incubated overnight at RT with the primary antibodies rabbit NTR1 (H-130); 1∶100 and goat Kit (C-14); 1∶500 (Santa Cruz Biotechnology, Inc., Santa Cruz, Ca), rinsed twice in TBS and incubated in the dark for 1 hour at RT in TBS containing the secondary antibodies anti rabbit biotinylated; 1∶200 (Jackson Immunoresearch, Cambridge, UK) and anti goat Alexa™ 448 conjugated; 1∶200 (Invitrogen, Eugene, Oregon). Slides were then rinsed twice in TBS and incubated in the dark for 1 hour at RT with streptavidin-coupled NorthernLight™ 557 (NL557) (R&D Systems, Abingdon, Oxon, UK) in TBS. Secondary antibodies anti rabbit DyLight™ 549 conjugated; 1∶200 (Jackson Immunoresearch, Cambridge, UK) were used for GIST882 cells immunolabeling.

The optimal working dilution was determined empirically for each antibody. The protocol used for double immunofluorescence staining did not modify the distribution or the intensity of each individual labeling observed in corresponding single procedures. Omission of the primary or of the secondary antibodies resulted in the absence of the respective fluorescent signal.

Nuclei were stained with 5 µM Hoechst in Tris-HCl (Merck-Belgolabo, Overijse, Belgium) 0.05M (pH 7.4), containing 0.5 mg/ml type 1-AS Ribonuclease A for 5 min in the dark at RT.

After three rinses in TBS, preparations were mounted with Fluor Save™ Reagent anti-fade mounting medium (Calbiochem, Nottingham, UK).

#### Confocal microscopy

Preparations were viewed under a LSM510 NLO multiphoton confocal microscope fitted on an Axiovert M200 inverted microscope equipped with a C-Apochromat 40×/1.2 N.A. water immersion objective (Zeiss, Iena, Germany).

The 543 nm excitation wavelength of the HeNe1 laser, a main dichroic HFT 488/543/633 and a long-pass emission filter (LP560 nm) were used for selective detection of the red fluorochrome. The nuclear stain Hoechst was excited in multiphotonic mode at 760 nm with a Mai Tai™ tunable broad-band laser (Spectra-Physics, Darmstad, Germany) and detected using a main dichroic HFT KP650 and a band-pass emission filter (BP435–485 nm).

Single optical sections, 2 µm thick, were collected for each fluorochrome sequentially. The images generated (pixel size: 0.1 µm) were merged and analyzed with the Zeiss LSM510 software and exported in .jpg image format.

#### Epifluorescence microscopy

Slides were examined under an AxioImager Z1 (Zeiss, Oberkochen, Germany) equipped with a Plan-Neofluar 40×/0.75 N.A. dry objective and band-pass filter sets nr 38, 15 and 49 for green, red and blue fluorochromes, respectively, using AxioVision software (Zeiss, Oberkochen, Germany).

### Human GIST tissue micro array (TMA)

We examined NTSR1-ir in 2 independent cohorts of GIST using tissue microarray slides of formalin-fixed, paraffin-embedded (FFPE) material. As the clinico-pathological characteristics available for the two cohorts had not been recorded in a uniform way, they were analyzed separately.

For the Cleveland Clinic GIST TMA, a cohort of human GIST samples was retrieved from the anatomic pathology files of the Cleveland Clinic. The collection and analysis of human tissue samples was approved by the Cleveland Clinic Institutional Review Board. GIST samples were used to construct a tissue microarray with 1 mm cores using a TMarrayer™ (P/N 02110016) semi-automated tissue arrayer (Pathology Devices, Westminster, MD, USA) Risk assessment was performed using the criteria of Miettinen and Lasota [17] as recommended by the National Cancer Care Network [18]. Mutations in *KIT* exons 9, 11, 13, and 17 and *PDGFRA* exons 12 and 18 were examined as described previously [5]. Each case was present in duplicate on in this array. Clinico-pathological characterization of GIST used for Cleveland Clinic GIST TMA is summarized in Table 2.

**Table 2:** Clinicopathologic features of the Cleveland Clinic GIST TMA.

**Primary tumor site**	**Total (n)**
Gastric	34
Small bowel	9
Colon	2
Rectovaginal	1
Disseminated	3
**Tumor morphology**	**Total (n)**
Spindle	35
Epithelioid	14
**Risk category**	**Total (n)**
Malignant	7
High risk	10
Moderate risk	13
Low risk	8
Very low risk	6
No risk	5
**Mitotic Figures**	**Total (n)**
≤5/50 HPF	26
>5/50 HPF	17
N.A.	6
**Mutation Status**	**Total (n)**
KIT mutation	25
KIT Exon 9 AY 502-503 duplication	4
KIT Exon 11 duplication	3
KIT Exon 11 deletion	6
KIT Exon 11 deletion - MYEV 552–555	1
KIT Exon 11 deletion - PMYE 551–555	1
KIT exon 11 V559D	6
KIT exon 11 V560D	1
KIT Exon 11 V600D	1
KIT exon 11 W557G	1
KIT Exon 13 K642E	1
PDGF mutation	9
PDGFRA Exon 12 - V561D	2
PDGFRA exon 12 deletion	1
PDGFRA Exon 18 D842V	4
PDGFRA Exon 18 D842Y	1
PDGFRA Exon 18 Ins-Del	1
Wild type	11
N.A.	4
**KIT-ir**	**Total (n)**
positive	44
negative	4
N.A.	1

Footnote: N.A.: data not available

SuperBiochips GIST TMA was purchased from Super BioChips laboratories, (#ADD1 , Seoul, South Korea). It contained 48 formalin-fixed and paraffin-embedded human GIST tissue specimens and 9 matched normal gut tissue specimens. According to the manufacturer, specimens (2 mm cores) were prepared from surgical specimens of American patients with GIST under guarantee in US law. Risk assessment was performed according to Fletcher et al. [3]. The clinico-pathological characterization of this GIST cohort is summarized in Table 3.

**Table 3:** Clinicopathologic features of the SuperBiochips GIST TMA.

**Sex/Age average**	**Total (n)/years**
Male	28/58.3
Female	20/62.6
**Primary tumor site**	**Total (n)**
Gastric	22
Small bowel	15
Abdominal cavity	1
Rectum	2
Disseminated	8
**Risk category**	**Total (n)**
Malignant	8
High risk	24
Intermediate risk	8
Low risk	8
**Mitotic Figures**	**Total (n)**
≤5/50	22
>5/50	26
**KIT-ir**	**Total (n)**
positive	48
negative	0

#### Immunohistochemistry (IHC) on FFPE human material

After rehydration through phenol and graded alcohols, TMA slides were heated at 96°C in 10mM Citrate buffer (pH 6.0) for 20 min to achieve epitope unmasking. Slides were then cooled for 30 min then rinsed in TBS. The staining with primary NTSR1 (C-20); 1∶50 (Santa Cruz Biotechnology, Inc., Santa Cruz, Ca), Kit/CD117; 1∶500 (Dako North America, Inc., Carpinteria, CA) antibodies and secondary anti-rabbit or anti-goat biotinylated antibodies; 1∶200 (Jackson Immunoresearch, Cambridge, UK), respectively, was performed as mentioned above for IF. Then sections were incubated in ABC solution (ABC kit standard PK-4000; Vector Laboratories, Burlingame, CA) for 90 min at room temperature and peroxidase activity revealed for 5–10 min with nickel-enhanced DAB (DAB-Ni), resulting in a black precipitate. The DAB-Ni solution was prepared by dissolving 0.3 g of nickel ammonium sulfate (Fluka, Buchs, Switzerland) and 10 mg of DAB (Sigma, St. Louis, MO) in 50 ml of 0.05 M Tris/HCl, pH 7.6. Immediately before use, 5 µl of 30% H_2_O_2_ (Merck, Darmstadt, Germany) was added.

Staining was regarded as positive when specific (i.e. above background) NTSR1-ir signal was present in ≥10% of tumor cells. The pattern of subcellular distribution of NTSR1-ir was recorded as: nuclear (without distinct cytoplasmic staining), cytoplasmic (without distinct nuclear staining), or mixed. χ^2^ (chi-squared), Fisher's exact test and Student's t-test, were used to test possible association between Ntsr1-ir and the clinico-pathological features of the tumors.

### Human GIST882 cell line

The human GIST cell line GIST882 [19] was kindly provided by Dr. Jonathan A. Fletcher, Harvard Medical School, Boston, Massachusetts, USA. Cells were cultured at 37°C in DMEM (GIBCO, California USA) supplemented with 10% fetal bovine serum (FBS), 2% Penicillin/Streptomycin. Cells were starved overnight in minimal medium (i.e. without FBS), before stimulation with the NTSR1 agonist JMV449 (10 µM) (Tocris Bioscience, Ellisville, MO) for 4 hours.

#### Western blotting (WB)

Cells were lysed in buffer containing 10 mM Tris-HCl (pH 7.5), 150 mM KCl, 100M NaF, 0.5% Nonidet P-40, 12mM B-mercaptoethanol and 20 µl/ml proteases inhibitor mixture (Roche; Mannheim, Germany). Proteins were solubilized in sample buffer, heated at 95°C for 5 min, separated by SDS-PAGE on 8% polyacrylamide gel and transferred on a 0.2 µm nitrocellulose membrane. Primary anti-NTSR1 antibody (C-20); 1∶100 (Santa Cruz Biotechnology, Inc., Santa Cruz, CA), secondary anti-goat IgG DyLight800 antibody; 1∶10000 (Pierce, Thermo Fisher Scientific, Erembodegem, Belgium) and the Odyssey™ imaging system (LI-COR Biotechnology, Lincoln, NE) was used to reveal the signal.

## Results

### Ntsr1 expression in murine Kit^K641E^ GIST model

#### Ntsr1 mRNA expression is up-regulated in KitK641E antrum

**Figure 1:**
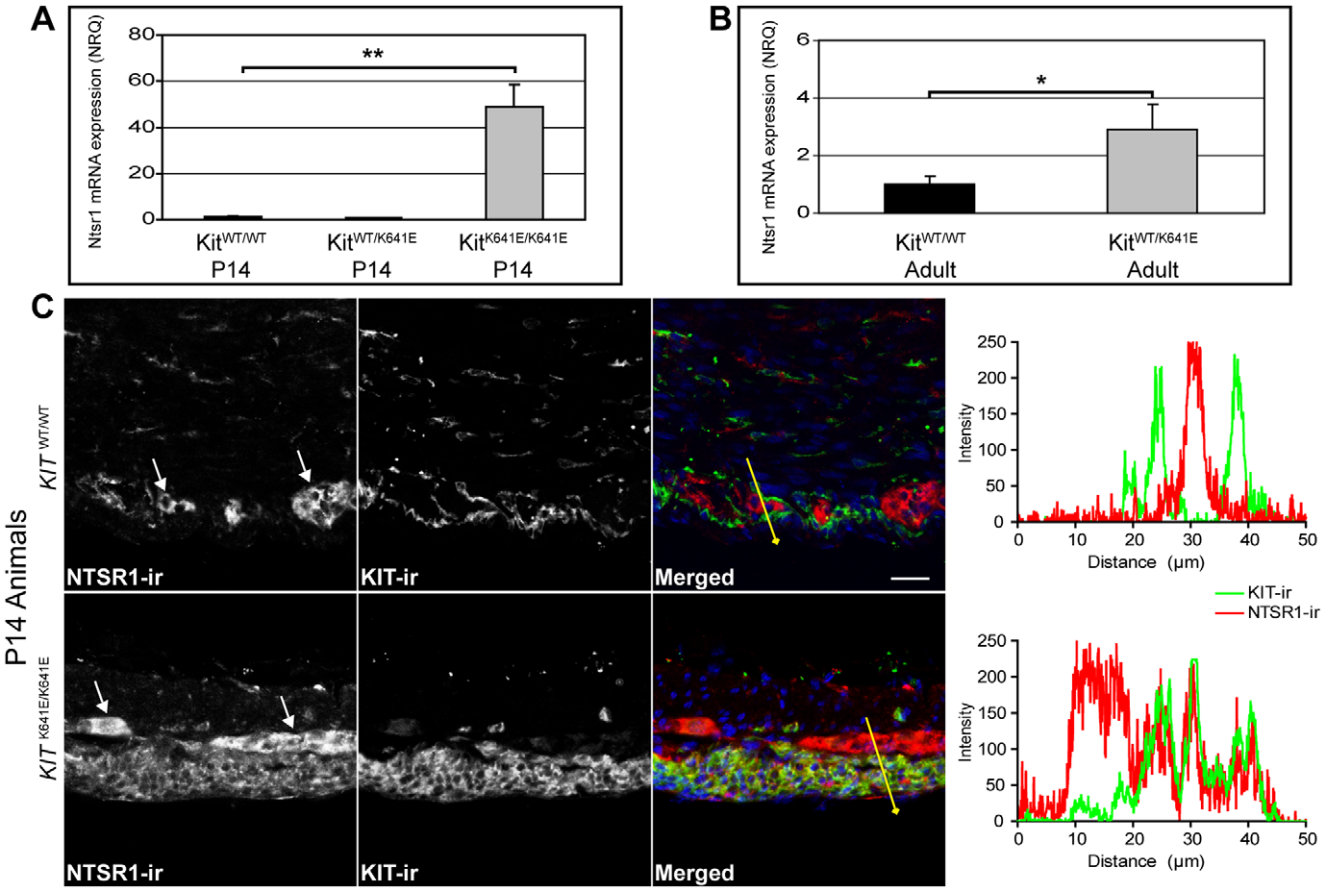
Ntsr1-ir is expressed in the hyperplastic layer of Kit-ir cells in Kit^K641E^ antrum but not in WT ICC. **A**: In P14 antrum, *Ntsr1* mRNA exhibited significant upregulation in *Kit* ^K641E/K641E^ homozygous compared to *Kit* ^WT/WT^ littermates. Ntsr1 expression in heterozygous *Kit* ^WT/K641E^ animals did not differ significantly from their *Kit* ^WT/WT^ littermates. Three animals of each genotype were used for quantification. Data are reported as the mean±SEM and presented as normalized relative quantities (NRQ), (**) p<0.005. **B**: In adult antrum, expression of *Ntsr1* mRNA was moderately increased in *Kit* ^WT/K64iE^ heterozygous compared to *Kit* ^WT/WT^ littermates. Three animals of each genotype were used for quantification. Data are reported as the mean±SEM and presented as normalized relative quantities (NRQ), (*) p<0.05. **C**: Representative single confocal images of Ntsr1-ir distribution in the P14 mouse antrum: Ntsr1-ir (NL559, red) was observed in the Kit-ir (Alexa 488, green) hyperplastic layer of P14 homozygous *Kit* ^K641E/K641E^ antrum (lower row) but was not present in the Kit-ir ICC in WT littermates (upper row). Ntsr1-ir was also consistently found in the myenteric plexus (white arrows) and intramuscular nerve fibers in all genotypes. Scale bar: 20 microns. Fluorescence intensity plots along the yellow lines illustrate the respective distribution of Ntsr1-ir (NL559, red) and Kit-ir (Alexa 488, green).

Relative expression of *Ntsr1* mRNA was markedly increased in *Kit* ^K641E/K641E^ homozygous P14 antrum compared to *Kit* ^WT/WT^ littermates (NRQ = 48.7; p = 7.7e-6) while Ntsr1 expression in heterozygous *Kit* ^WT/K641E^ P14 animals was similar to their WT *Kit* ^WT/WT^ littermates (Fig. 1A). *Kit* ^K641E/K641E^ homozygous animals usually die before weaning. In the antrum of adult mice, the relative increase of *Ntsr1* expression in *Kit* ^WT/K641E^ heterozygous compared to *Kit* ^WT/WT^ littermates was moderate, but significant (NRQ = 2.9; p = 0.049) (Fig. 1B).

*Ntsr2* transcript was detected neither in *Kit* ^K641E/K641E^ homozygous P14 antrum nor in *Kit* ^WT/WT^ littermates. Relative *Ntsr3* mRNA expression did not differ in *Kit* ^K641E/K641E^ homozygous P14 antrum compared to *Kit* ^WT/WT^ littermates (p = 0.33) (data not shown).

#### Ntsr1-ir is not present in WT ICC

Ntsr1-ir was observed in the myenteric plexus and intramuscular nerve fibers in P14 *Kit* ^WT/WT^ (Fig. 1C upper row), as well as in adult *Kit* ^WT/WT^ animals (Fig. S1). Conversely, ICC, identified by Kit-ir, were consistently negative for Ntsr1-ir in WT animals.

#### Ntsr1-ir is expressed in the hyperplastic layer of Kit-ir cells in KitK641E antrum

In the antrum of P14 *Kit* ^K641E/K641E^ animals, Ntsr1-ir decorated the Kit-ir hyperplastic layer (Fig. 1C bottom row), while in P14 *Kit* ^WT/K641E^ heterozygous antrum, Ntrs1-ir did not differ from P14 WT animals (data not shown). In adult heterozygous *Kit* ^WT/K641E^ animals, Ntsr1-ir was detected also in hyperplastic clusters of Kit-ir cells but not in individual Kit-ir ICC without sign of hyperplasia (Fig. S1). In all genotypes, myenteric plexus and intramuscular nerve fibers consistently exhibited Ntsr1-ir.

### NTSR1 expression in human GIST

#### NTSR1-ir is not present in human KIT-ir ICC

**Figure 2:**
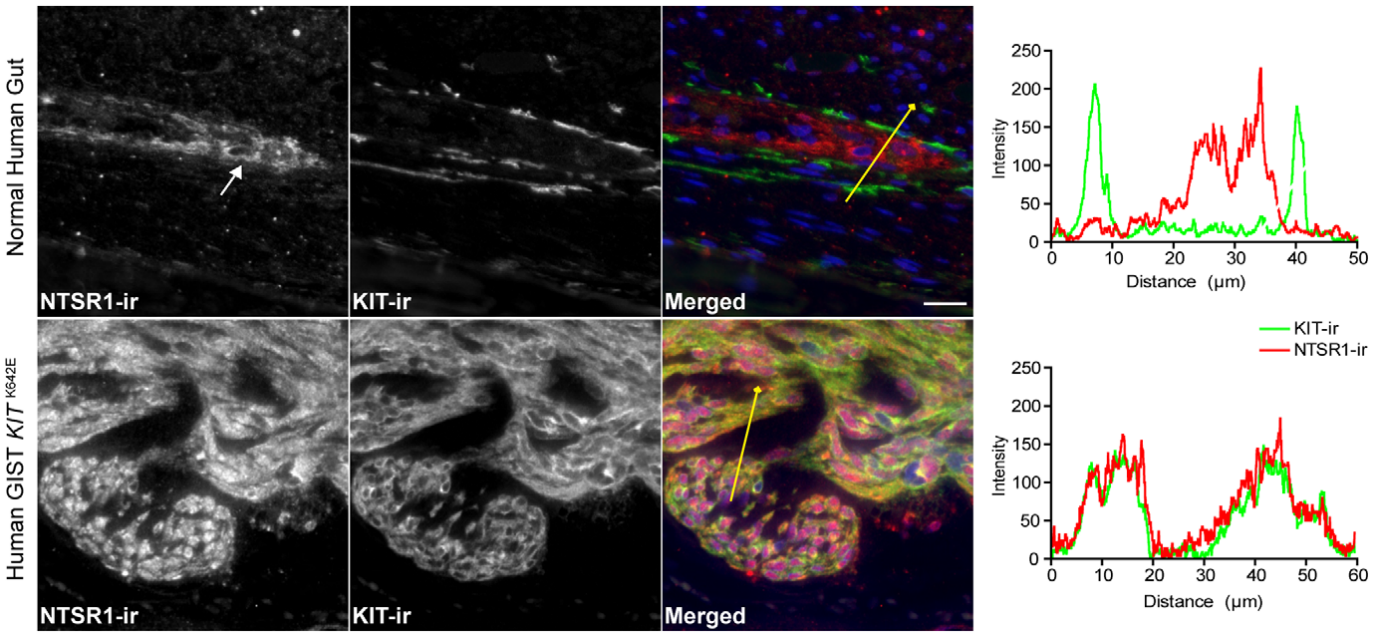
NTSR1-ir is present in a human GIST with KIT K642E mutation but not in normal human KIT-ir ICC. Representative epifluorescence microscopy images of NTSR1-ir distribution in the human gut: In the normal human colon, NTSR1-ir (NL559, red) was detected in myenteric plexus (white arrows) but not in the adjacent KIT-ir (Alexa 488, green) ICC (upper row). In a human GIST with *KIT* K642E mutation, NTSR1-ir colocalized with KIT-ir in the tumor cells (lower row). Scale bar: 20 microns. Fluorescence intensity plots along the yellow lines illustrate the respective distribution of NTSR1-ir (NL559, red) and KIT-ir (Alexa 488, green).

In the normal human colon, NTSR1-ir was detected in MP but not in the adjacent KIT-ir ICC (Fig. 2 upper row). Omission of primary antibody completely wiped out the signal and background (non-specific) signal in other cell types was minimal.

In a human GIST with *KIT* K642E mutation, NTSR1-ir was present only in the KIT-ir tumor cells (Fig. 2 bottom row).

#### NTSR1-ir is present in human GIST irrespective to the mutation status

Immunohistochemistry for NTSR1 was performed on two independent TMA including a total of 97 GIST. 95 samples were considered for analysis, 2 spots were excluded due to poor quality of tissue. Clinico-pathological characteristics of the GIST cases are summarized in Tables 2 and 3.

**Figure 3:**
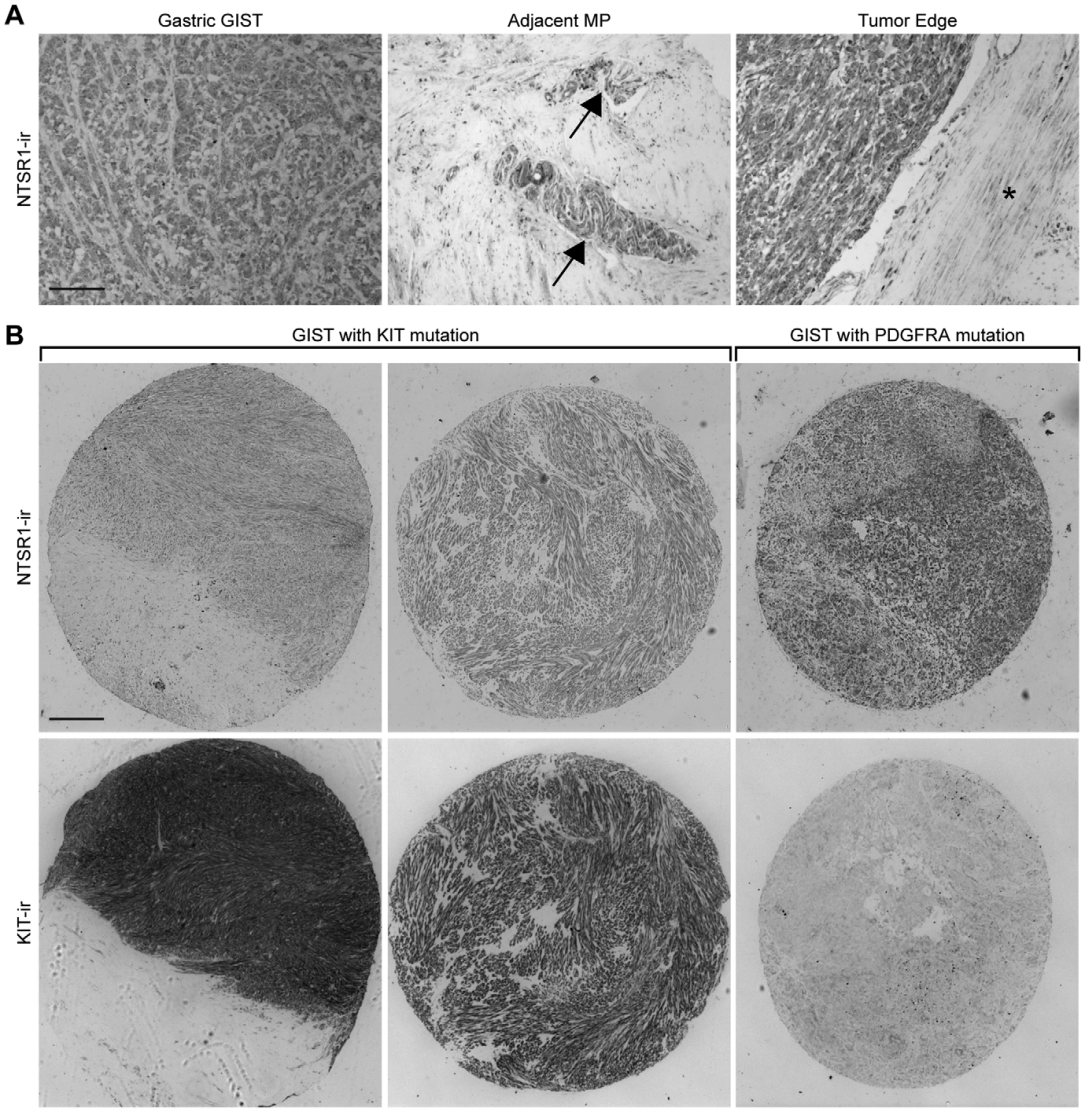
NTSR1-ir in human GIST is irrespective to the mutation status. NTSR1 and KIT immunohistochemistry on FFPE human TMA. **A**: Representative example of NTSR1-ir in a gastric GIST (left). NTSR1-ir was also present in adjacent myenteric plexus (arrows) (middle) while adjacent non tumoral tissue (*) (right) exhibited only minimal background (non specific) signal. **B**: Column 1 and 2: Representative examples of NTSR1-ir in KIT-ir GIST while (KIT negative) non-tumoral tissue exhibited only minimal (non specific) background signal. Column 3: Example of a KIT negative GIST with PDGFRA mutation positive for NTSR1-ir. Scale bars in A and B: 20 microns.

All GIST stained positively for NTSR1-ir (Fig. 3A left and Fig. 3B). In adjacent non-tumoral tissue, NTSR1-ir was present in myenteric plexus but not in other cell types (Fig. 3A middle and right, respectively). Background (non specific) signal in non-tumoral tissue was minimal, confirming the specificity of the NTSR1-ir signal observed on TMA.

Noteworthy, 4 KIT negative GIST with *PDGFRA* mutation were positive for NTSR1 staining (Fig. 3B). All 4 cases were of gastric origin, 3/4 showed epithelioid morphology and belong to the low-moderate risk category (Table 4).

**Table 4:** Clinicopathologic characteristics of NTSR1 positive/KIT negative GIST specimens.

No	Morphology	Primary site	Risk Assessment	Mutation Status	Size	Mitotic Figures	NTSR1 -ir
1	Epithelioid	gastric	moderate	PDGFRA Exon 12	13 cm	4/50 HPH	mix
2	Epithelioid	gastric	low	PDGFRA Exon 18	5.2 cm	2/50 HPH	mix
3	Epithelioid	gastric	moderate	PDGFRA Exon 12	15 cm	1/50 HPH	nuclear
4	Spindle	gastric	moderate	PDGFRA Exon 18	17 cm	2/50 HPH	nuclear

**Figure 4:**
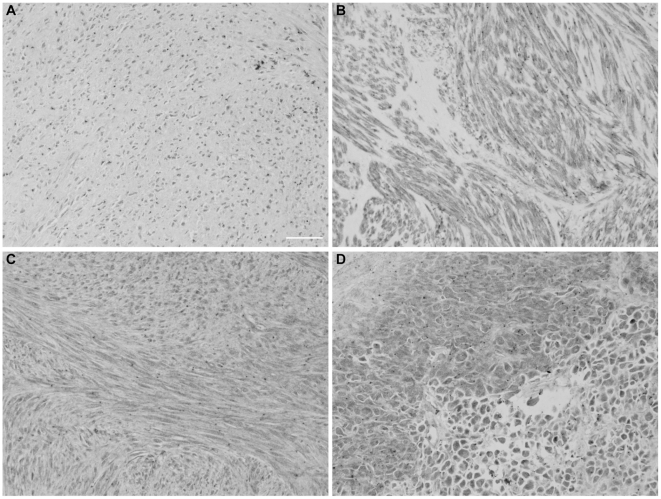
NTSR1-ir patterns in human GIST. Representative images of NTSR1-ir staining patterns encountered on GIST TMA: **A**: Nuclear staining. **B**: Cytoplasmic staining. **C** Mixed pattern: NTSR1-ir present in both nucleus and cytoplasm of the tumor cells. **D**: Mixed pattern: nuclear and cytoplasmic NTSR1-ir patterns coexisting in adjacent parts of the tumor. Scale bar: 20 microns.

Three patterns of NTSR1-ir were observed (Fig. 4): nuclear, without distinct cytoplasmic staining (Fig. 4A); cytoplasmic, without distinct nuclear staining (Fig. 4B); and mixed staining pattern, with NTSR1-ir present in nucleus as well as in cytoplasm (Fig. 4C). Mixed and nuclear staining were occasionally encountered in close vicinity (Fig. 4D) and were regarded as mixed patterns. A majority of GIST (63/95) showed mixed (nuclear + cytoplasmic) NTSR1-ir. The pattern of NTSR1-ir was exclusively cytoplasmic in 25/95 samples and exclusively nuclear in 7/95 (Tables 5 and 6).

**Table 5:** NTSR1-ir on the Cleveland Clinic GIST TMA.

Clinicopathologic features	NTSR1-ir (n/total)		
	Nuclear	Cytoplasmic	Mix
**Primary tumor site**			
Gastric	2/34	7/34	24/34
Small bowel	1/9	1/9	6/9
Colon	1/2	0/2	1/2
Rectovaginal	0/1	1/1	0/1
Disseminated	0/3	1/3	2/3
**Tumor morphology**			
Spindle	4/35	7/35	22/35
Epithelioid	0/14	3/14	11/14
**Risk category**			
Malignant - High	2/17	4/17	11/17
Moderate - Low	2/21	5/21	14/21
Very low - None	0/11	1/11	8/11
**Mitotic Figures**			
≤5/50	3/26	2/26	10/26
>5/50	1/17	7/17	18/17
**Mutation Status**			
KIT mutation	3/25	7/25	15/25
KIT Exon 9 AY 502-503 duplication	2/4	0/4	2/4
KIT Exon 11 duplication	0/3	0/3	2/3
KIT Exon 11 deletion	1/6	3/6	2/6
KIT Exon 11 deletion - MYEV 552–555	0/1	0/1	1/1
KIT Exon 11 deletion - PMYE 551–555	0/1	1/1	0/1
KIT exon 11 V559D	0/6	2/6	4/6
KIT exon 11 V560D	0/1	0/1	1/1
KIT Exon 11 V600D	0/1	0/1	1/1
KIT exon 11 W557G	0/1	0/1	1/1
KIT Exon 13 K642E	0/1	1/1	1/1
PDGF mutation	0/9	3/9	6/9
PDGFRA Exon 12 - V561D	0/2	1/2	1/2
PDGFRA exon 12 deletion	0/1	0/1	1/1
PDGFRA Exon 18 D842V	0/4	2/4	2/4
PDGFRA Exon 18 D842Y	0/1	0/1	1/1
PDGFRA Exon 18 Ins-Del	0/1	0/1	1/1
Wild type	1/11	0/11	10/11

**Table 6:** NTSR1-ir on the SuperBiochips GIST TMA.

Clinicopathologic features	NTSR1-ir (n/total)		
	Nuclear	Cytoplasmic	Mix
**Sex**			
Male	2/28	8/28	18/28
Female	1/20	7/20	12/20
**Primary tumor site**			
Gastric	2/22	7/22	13/22
Small bowel	0/15	5/15	10/15
Abdominal cavity	0/1	0/1	1/1
Rectum	1/2	1/2	0/2
Disseminated	0/8	2/8	6/8
**Risk category**			
Malignant	0/8	2/8	6/8
High risk	2/24	8/24	14/24
Intermediate risk	1/8	1/8	6/8
Low risk	0/8	4/8	4/8
**Mitotic Figures**			
≤5/50	0/22	8/22	14/22
>5/50	3/26	7/26	16/26

Altogether, no statistically significant correlation could be found between NTSR1-ir and the GIST clinico-pathological features.

### NTSR1 - but not Neurotensin (NT) - is expressed in the human GIST882 cell line

In the human GIST882 cell line, NTSR1 expression was confirmed by RT-PCR (Fig. S2A). and WB (Fig. S2B) while transcript of neurotensin/neuromedin N precursor (NT/N) was not detected by RT-PCR. The human astrocytoma cell line 1321N1 served as positive control (Fig. S2A).

#### NTSR1-ir translocates to the nucleus upon agonist stimulation in GIST882 cells

After overnight starvation in FBS free medium (to avoid any NT that might be present in FBS), GIST882 cells exhibited diffuse perinuclear cytoplasmic, as well as nuclear, NTSR1-ir. Application of the NTSR1 agonist JMV449 (10 µM) for 4 hours markedly enhanced nuclear NTSR1-ir and redistributed cytoplasmic NTSR1-ir into dot-like clumps (Fig. S2C).

## Discussion

To the best of our knowledge, the distribution of NTSR1-ir in the muscularis propria of the gut had not been previously reported. We observed NTSR1-ir in the soma of myenteric neurons and in nerve fibers in the muscle layers, in both mouse and human gut, while the KIT-ir ICC and other cell types were not labeled.

Conversely, NTSR1-ir was consistently detected in the hyperplastic Kit-ir cells in the murine *Kit* ^K641E^ GIST model and in all 95 human GIST investigated, irrespective of the presence of KIT-ir or KIT/PDGFRA mutation. The expression of NTSR1-ir in GIST, but not in normal ICC, contrasts with other markers recently reported in human GIST, which are generally also expressed in normal ICC [7], [10], [11].

We have identified three distinctive patterns of NTSR1-ir (nuclear; cytoplasmic; mixed) in GIST TMA but they could not be correlated with the recorded histopathological characteristic of the tumors. Nuclear localization of NTSR1 has been linked to cell responses evoked by intense and persistent agonist stimuli. [20]–[23]. NT, the natural NTSR1 high-affinity ligand, is a gut hormone normally present in serum and surges upon feeding [24], [25]. In tissues, NT can also be produced by tumoral cells [26] or by reactive cells in their vicinity [22], [27]–[30], creating autocrine or paracrine activating loops, respectively. Hence, the variable proportions of nuclear and cytoplasmic NTSR1-ir observed in GIST TMA, possibly represents different levels of NTSR1 activation caused by a complex and dynamic interplay of various sources of ligand and possibly other factors. The precise origin of NTSR1 activation in individual GIST remains to be established. In the GIST882 cell line, the redistribution of NTSR1-ir upon stimulation by the specific NTSR1 agonist JMV499 suggests the presence of functionally responsive NTSR1 while the absence of NT/N precursor mRNA expression rule out an autocrine activation loop in that cell line.

High affinity NT binding sites have been found in various cancers, including Ewing's sarcoma [31] and NTSR1 expression, driven by the Wnt/b-catenin pathway, is an early event in oncogenic transformation [22], [23]. Sustained activation of NTSR1 leads to persistent activation of signaling pathways, including ERK1/2 [20], hence facilitating tumor growth and progression. NTSR1 has been associated with progression in lung, colon, pancreas, prostate and breast epithelial cancers [29], [30], [32]. According to the Gene Expression Omnibus (GEO) database (http://www.ncbi.nlm.nih.gov/geo/), NTSR1 is also expressed in a variety of sarcoma. Therefore, NTSR1 expression appears to be linked to tumorigenesis in general and cannot be used as a diagnostic tool to distinguish GIST from other tumor types or to predict their risk of malignancy.

Nevertheless, the identification of NTSR1 expression in GIST but not in the KIT expressing ICC cells from which they derive, offers clinical perspectives that deserve further consideration.

Firstly, NTSR1 can be targeted with radio-labeled NT analogues for whole body tumor imaging and possibly even for subsequent therapeutic interventions. Currently, 18F-Fluoro-deoxyglucose (18F-FDG) positron emission tomography (PET) is used for assessing tumor response to tyrosine kinase inhibitors in vivo [33]–[35]. However, 18F-FDG-PET cannot differentiate between GIST and other gastrointestinal malignancies including epithelial cancers and lymphoma and in some cases, 18F-FDG-PET fails to detect the relapse of GIST at an early time point [36]. An analogue based on NT (8–13) NT-XIX, has already been synthesized and characterized. Radioactivity clearance of NT-XIX analogue from healthy organs was faster than from tumors, resulting in improved tumor-to-tissue ratios and good SPECT/CT imaging [15]. NTSR1 in vivo imaging has already been successfully used in patients with ductal pancreatic adenocarcinoma [37]. Therefore NTSR1 in vivo imaging deserves full consideration in GIST.

NTSR1 may also be considered as a prospective direct therapeutic target. NT antagonists, such as SR48692.21, counteract the stimulatory effect of NT on tumor cells [22]. The influence of SR48692.21 on GIST proliferative characteristics thus deserves consideration.

Finally, NTSR1 could represent the gateway to introduce cytotoxic chemicals or expression vectors targeting the constitutively activated pathways into the cancerous cells [38]–[40].

In conclusion, further studies are needed to substantiate the potential of NTSR1 for clinical interventions in GIST.

## Supporting Information

Figure S1 Ntsr1-ir is expressed in Kit-ir cell clusters in the antrum of adult heterozygous KitWT/K641E mice but not in WT ICC. Representative epifluorescence images of Ntsr1-ir distribution in the adult mouse antrum: Ntsr1-ir (NL559, red) was observed in Kit-ir (Alexa 488, green) cell clusters (insert) in adult heterozygous KitWT/K641E mice, but not in WT littermates. Ntsr1-ir was also consistently found in the myenteric plexus (*) and intramuscular nerve fibers both genotypes. Scale bars: 20 microns.
(4.35 MB TIF)

Figure S2 NTSR1 is expressed in the human GIST882 cell line and responds to agonist stimulation. A: NTSR1 - but not neurotensin/neuromedin N precursor (NT/N) mRNA - is expressed in GIST882 cells. Lane 1: GIST882 cDNA, lane 2: 1321N1 cDNA, lane 3: negative control (no template). RT-PCR revealed the presence of NTSR1 amplicon (90bp) but not NT/N (121bp) in GIST882 cells while 1321N1 express both NTSR1 and NT/N. β-ACTIN (213bp) was used as control. B: WB for NTSR1 in GIST882 cells. A single band of the expected MW (54kDa) was detected. C: Representative confocal microscopy images of NTSR1-ir patterns in GIST882 cells. Upper row: After overnight incubation in FBS free medium, a diffused cytoplasmic and nuclear NTSR1-ir (DyLight 549, red) was observed. Lower row: After stimulation with the NTSR1 agonist JMV449 for 4 hours, NTSR1-ir strongly increased in the nucleus and in many cells, cytoplasmic NTSR1-ir exhibited a dot-like pattern. Scale bar: 20 microns. Fluorescence intensity plots along the yellow lines illustrate the respective distribution of NTSR1-ir (DyLight 549, red) and DNA (Hoechst, blue).
(9.69 MB TIF)
