# Supplementary figures and images for: Neurotensin Receptor 1 Is Expressed in Gastrointestinal Stromal Tumors but Not in Interstitial Cells of Cajal
Source: PLoS One. 2011 Feb 18;6(2):e14710. doi: 10.1371/journal.pone.0014710 (PMC3041753; doi:10.1371/journal.pone.0014710)

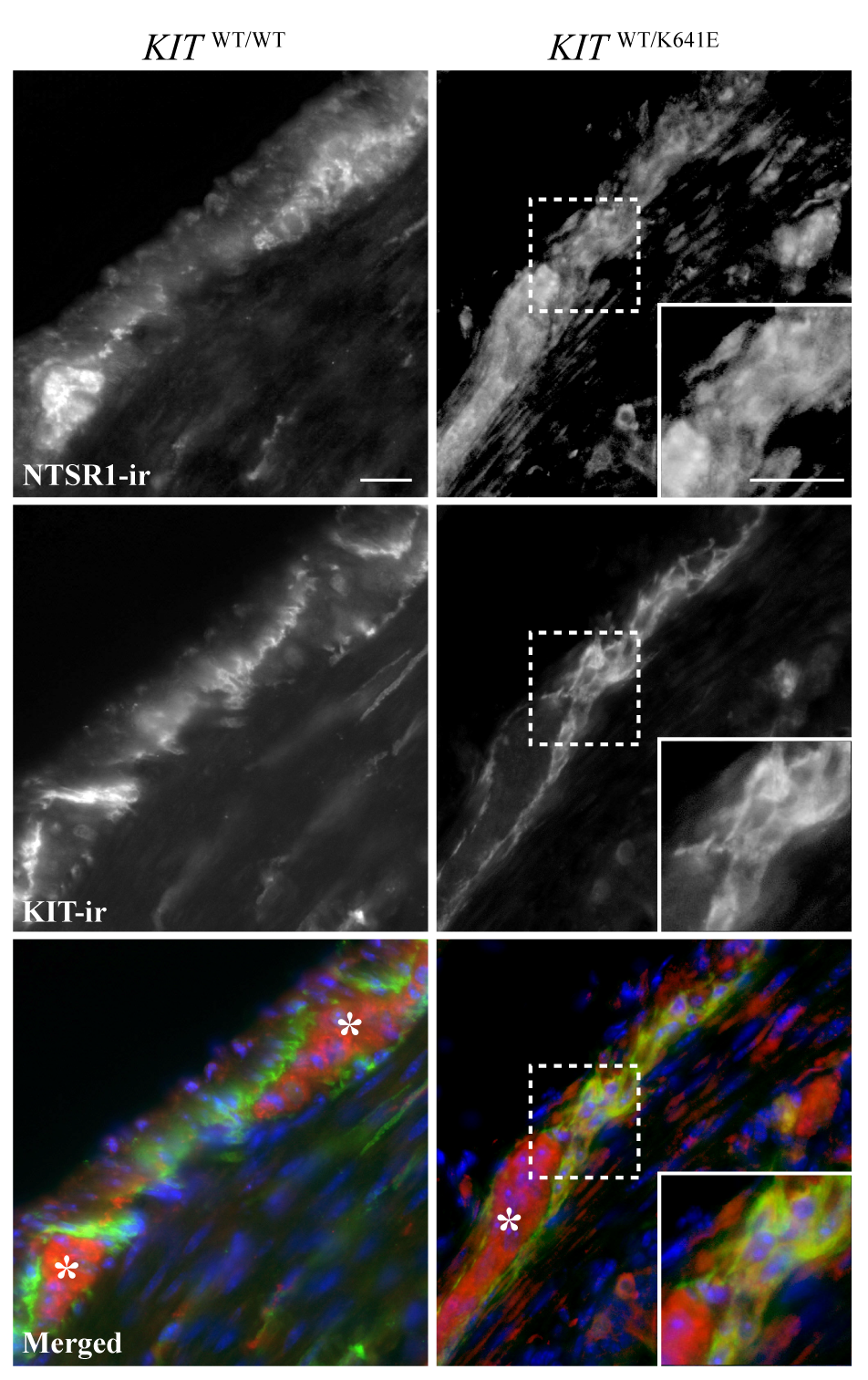

Supplement: Figure S1 — Ntsr1-ir is expressed in Kit-ir cell clusters in the antrum of adult heterozygous KitWT/K641E mice but not in WT ICC. Representative epifluorescence images of Ntsr1-ir distribution in the adult mouse antrum: Ntsr1-ir (NL559, red) was observed in Kit-ir (Alexa 488, green) cell clusters (insert) in adult heterozygous KitWT/K641E mice, but not in WT littermates. Ntsr1-ir was also consistently found in the myenteric plexus (*) and intramuscular nerve fibers both genotypes. Scale bars: 20 microns. (4.35 MB TIF) [file pone.0014710.s001.tif]

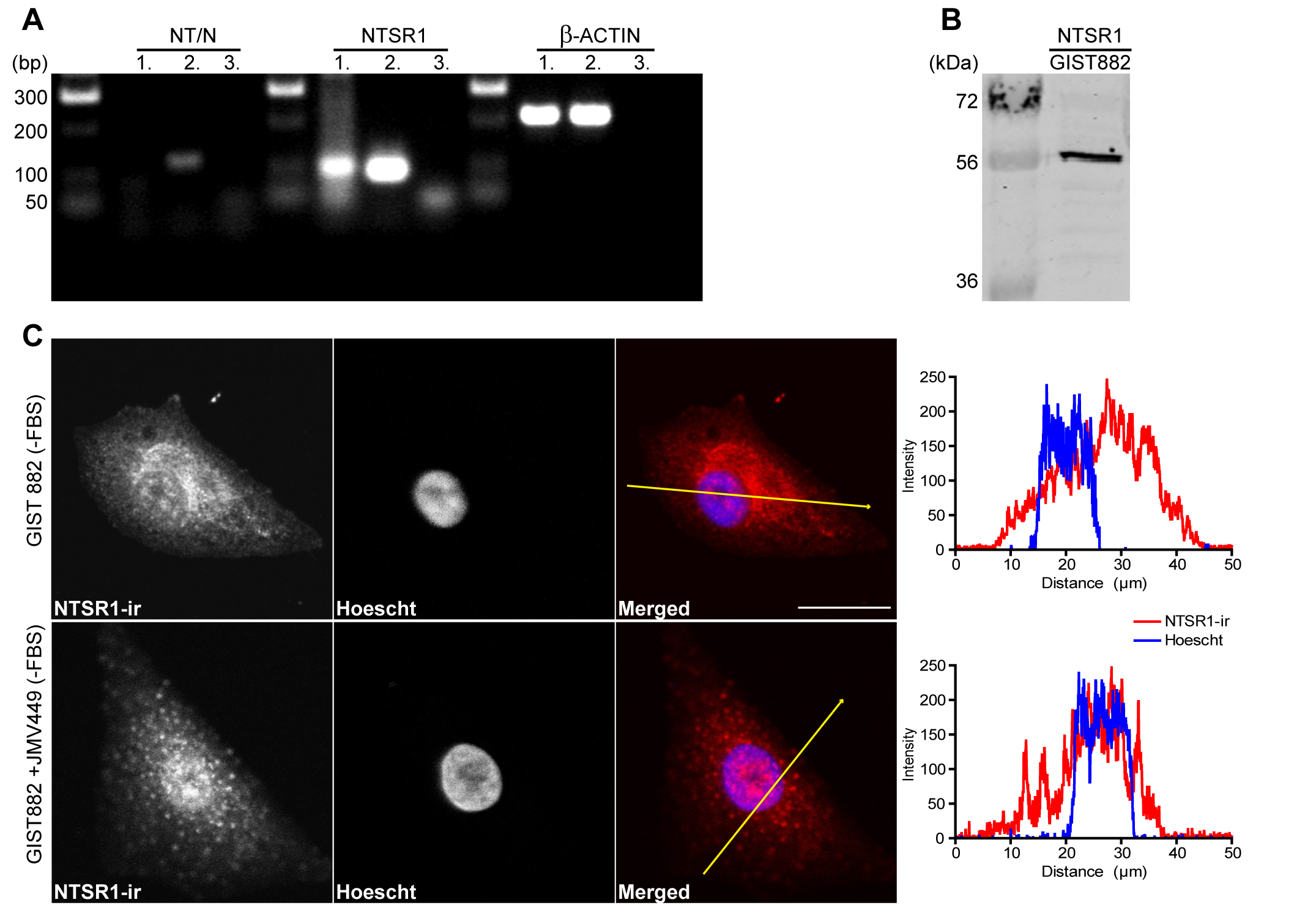

Supplement: Figure S2 — NTSR1 is expressed in the human GIST882 cell line and responds to agonist stimulation. A: NTSR1 - but not neurotensin/neuromedin N precursor (NT/N) mRNA - is expressed in GIST882 cells. Lane 1: GIST882 cDNA, lane 2: 1321N1 cDNA, lane 3: negative control (no template). RT-PCR revealed the presence of NTSR1 amplicon (90bp) but not NT/N (121bp) in GIST882 cells while 1321N1 express both NTSR1 and NT/N. β-ACTIN (213bp) was used as control. B: WB for NTSR1 in GIST882 cells. A single band of the expected MW (54kDa) was detected. C: Representative confocal microscopy images of NTSR1-ir patterns in GIST882 cells. Upper row: After overnight incubation in FBS free medium, a diffused cytoplasmic and nuclear NTSR1-ir (DyLight 549, red) was observed. Lower row: After stimulation with the NTSR1 agonist JMV449 for 4 hours, NTSR1-ir strongly increased in the nucleus and in many cells, cytoplasmic NTSR1-ir exhibited a dot-like pattern. Scale bar: 20 microns. Fluorescence intensity plots along the yellow lines illustrate the respective distribution of NTSR1-ir (DyLight 549, red) and DNA (Hoechst, blue). (9.69 MB TIF) [file pone.0014710.s002.tif]
